# Endovascular Therapy for Tandem Occlusion in Acute Ischemic Stroke: Intravenous Thrombolysis Improves Outcomes

**DOI:** 10.3390/jcm8020228

**Published:** 2019-02-10

**Authors:** Slaven Pikija, Jozef Magdic, Laszlo K. Sztriha, Monika Killer-Oberpfalzer, Nele Bubel, Anita Lukic, Johann Sellner

**Affiliations:** 1 Department of Neurology, Christian Doppler Medical Center, Paracelsus Medical University, 5020 Salzburg, Austria; s.pikija@salk.at (S.P.); n.bubel@salk.at (N.B.); 2 Department of Neurology, University Medical Center Maribor, Maribor 2000, Slovenia; jozef_magdic@yahoo.com; 3 Department of Neurology, King’s College Hospital, Denmark Hill, London SE5 9RS, UK; laszlo.striha@kcl.ac.uk; 4 Research Institute for Neurointervention, Christian Doppler Medical Center, Paracelsus Medical University, 5020 Salzburg, Austria; m.killer@salk.at; 5 Department of Anesthesiology, General Hospital Varazdin, 42000 Varazdin, Croatia; lukic.anita@yahoo.com; 6 Department of Neurology, Klinikum rechts der Isar, Technische Universität München, 81675 München, Germany

**Keywords:** ischemic stroke, tandem occlusion, endovascular therapy, intravenous thrombolysis

## Abstract

Ischemic stroke related to tandem internal carotid and middle cerebral artery (TIM) occlusion is a challenging condition where endovascular treatment (EVT) is an emerging revascularization option. The identification of factors influencing clinical outcomes can assist in creating appropriate therapeutic algorithms for such patients. This study aimed to evaluate prognostic factors in the context of EVT for TIM occlusion. We performed a retrospective study of consecutive patients with TIM occlusion admitted within 6 h from symptom onset to two tertiary stroke centers. We recorded the etiology of stroke, clinical deficits at stroke onset and discharge, details of EVT, final infarct volume (FIV), in-hospital mortality, and outcome at three months. Among 73 patients with TIM occlusion, 53 were treated with EVT. The median age was 75.9 years (interquartile range (IQR) 64.6–82.6), with the most common etiology of cardioembolism (51.9%). Intravenous thrombolysis with tissue-plasminogen activator (t-PA) was performed in the majority (69.8%) of cases. EVT achieved successful recanalization with a thrombolysis in cerebral infarction (TICI) grade of 2b or 3 in 67.9%. A good outcome (modified Rankin score of 0–2 at three months) was observed in 37.7%. After adjustment for age, the National Institutes of Health Stroke Scale (NIHSS) at admission, and success of recanalization, smaller final infarct volume (odds ratio (OR) 0.021 for FIV above 25th percentile (95% CI 0.001–0.332, *p* = 0.005)) and administration of intravenous t-PA (OR 12.04 (95% CI 1.004–144.392, *p* = 0.049)) were associated with a good outcome at three months. Our study demonstrates that bridging with t-PA is associated with improved outcomes in the setting of tandem ICA and MCA occlusions treated with EVT and should therefore not be withheld in eligible patients.

## 1. Introduction

Concomitant internal carotid artery (ICA) occlusion is detected in up to 25% of patients with acute middle cerebral artery (MCA) infarction [1,2,3]. Moreover, acute ICA occlusion is associated with proximal MCA occlusion in 50% of cases [3]. The natural course of this condition, termed “tandem internal carotid and middle cerebral artery” (TIM) occlusion, is often devastating, with several clinical studies reporting a mortality as high as 55% [4,5,6,7]. In one study, only 20% of patients had a good clinical outcome after treatment with recombinant tissue-plasminogen activator (t-PA) [8]. Additional studies confirmed low efficacy of intravenous or intra-arterial thrombolysis in TIM occlusion, which could be related to the length of the thrombus [9,10,11,12].

Endovascular treatment (EVT) is the method of choice when treating stroke associated with large vessel occlusion including those of the ICA or MCA. EVT using intracranial stent retrieval-assisted thrombectomy can be combined with extracranial stenting/dilatation in cases of TIM occlusion, where t-PA treatment alone would achieve recanalization in only 9% of patients [13,14]. Previous studies consistently demonstrated favorable outcomes defined as modified Rankin Score (mRS) of 0–2 in 28–35% of patients receiving EVT [15,16]. There may also be an increased risk of intracranial hemorrhage with the administration of combined antithrombotic medications after acute stenting in the setting of TIM occlusion [17]. 

In this study, we hypothesized that patients with TIM treated with EVT may benefit from adjunctive t-PA. We therefore characterized the association of a number of variables with clinical outcome at three months in the setting of TIM occlusion in patients treated with EVT, with or without t-PA. This information can be helpful in the future to identify patients more likely to have unfavorable outcomes, as well as aid clinical decision making. 

## 2. Experimental Section

We performed a retrospective review of all consecutive stroke patients admitted to Christian Doppler Medical Center (Salzburg, Austria) and University Medical Center Maribor (Slovenia). The study protocol was reviewed by the Ethics Committee of Bundesland Salzburg, who decided that, due to the retrospective study design, no further ethics review was necessary (415-EP/73/750-2017). 

The study period was 2012–2016. The inclusion criteria were acute anterior circulation ischemic stroke within 6 h of symptom onset, ≥18 years of age, CT-angiography (CTA) confirmed TIM occlusion defined as ICA occlusion (extra- or intracranial) combined with occlusion of the MCA (M1 or M2), with a plan to perform stent-retriever based EVT [18]. Details of the scanners and imaging protocols were reported previously [19]. 

Additional investigations included 24-h electrocardiography (ECG), transthoracic and, when needed, transesophageal cardiac ultrasound, neurovascular ultrasound of the extracranial and intracranial vessels, and autoimmune and vasculitic screens. The etiology of stroke was determined using the Trial of Org 10172 in Acute Stroke (TOAST) criteria [20]. Additional recorded parameters were the times for starting intravenous thrombolysis with t-PA and groin puncture for EVT. Success of EVT was rated according to the thrombolysis in cerebral infarction (TICI) score [21]. A TICI score of 0, 1 or 2a was defined as unsuccessful reperfusion, whereas those with scores of 2b or 3 were rated as successful. A repeat computer tomography / magnetic resonance imaging (CT/MRI) within 24 h after the procedure was used to determine the presence of symptomatic intracerebral hemorrhage (sICH). Follow-up CT scans performed between 24 h and 7 days were analyzed to determine the final infarct volume (FIV) using previously reported methodology [7,19]. Additional variables included demographic and laboratory data, as well as the National Institutes of Health Stroke Scale (NIHSS) on admission and at discharge. Structured telephone interviews were conducted by certified assessors to determine the mRS at 3 months, as per national clinical guidelines. The mRS at 3 months was the primary outcome measure, dichotomized as 0–2 (good outcome) or 3–6 (bad outcome).

### Statistical Analysis

Data on patient demographics were summarized using descriptive statistics. Depending on the normality of distribution (tested by Kolmogorov-Smirnov test), continuous variables were compared using either the t-test for independent samples, or the Mann-Whitney test. Categorical variables were compared using the Fisher’s exact test. We performed multivariate analyses using binary logistic regression to calculate odds ratios. Age and the NIHSS score at admission were entered as continuous variables, whereas the binary co-variables included FIV (as a continuous in the first model and as ≤25 or >25 percentile in the second model), success of recanalization, and use of intravenous t-PA. All statistical analyses were performed using R Software version 3.5.1 (R Core Team, General Public Licence, 2010) [22]. A *p* value of 0.05 was used as the threshold for statistical significance.

## 3. Results

We identified 73 patients with TIM occlusion, seven (10%) from the center in Maribor. Further analyses were performed on 53 (75%) who received EVT. Demographics are shown in Table 1. 

The median age was 75.9 years (interquartile range (IQR) 64.6–82.6), with women comprising 47.2%. The median NIHSS score at presentation was 20 (IQR 16–23). The M1 portion of the MCA was occluded in 51 patients, and an M2 occlusion was detected in two. EVT was successful in 67.9% of patients (TICI 2b and 3). Acute ICA stenting was performed in 13%. Intravenous t-PA was used in 70%. There were 15 (28%) hospital deaths. Symptomatic intracranial hemorrhage occurred in 28% of the patients, but none occurred in the cases with acute ICA stenting. 

A good outcome was achieved in 20 (38%) patients; this was associated with younger age, lower NIHSS score at discharge, lower blood glucose at admission, higher cholesterol and low-density-lipoprotein (LDL) levels, and higher erythrocyte count (Table 2). There was a trend for better outcomes with a non-cardioembolic etiology of stroke (*p* = 0.058). The administration of intravenous t-PA in addition to EVT was associated with a better outcome. Successful recanalization and a lower infarct volume were also predictors of a better level of functioning at three months. 

In the multivariable analysis adjusted for age, NIHSS at admission, FIV as continuous variable, and administration of intravenous t-PA, only FIV appeared to be an inverse prognosticator for a good outcome (OR 0.924, 95% CI 0.868–0.985, *p* = 0.016). However, when FIV is dichotomized into above 25% and below and equal to 25th percentile, the following variables emerged as significant predictors of a good outcome at three months: Increasing age (OR 0.889 per year, 95% CI 0.805–0.981, *p* = 0.020), smaller FIV (OR 0.021 for FIV above 25th percentile, 95% CI 0.001–0.332, *p* = 0.006), and administration of intravenous t–PA (OR 12.04, 95% CI 1.004–144.392, *p* = 0.049) (Table 3).

Patient selection for intravenous thrombolysis adhered to national guidelines. We found that patients undergoing thrombolysis were significantly younger (median 71.0 (IQR 65.0–83.0) vs. 82.0 (77.0–85.5) years, *p* = 0.011) and had significantly lower NIHSS at admission (median 18 (14.5–22.0) vs. 23.0 (21.5–23.0), *p* = 0.01). The cohort of patients who received t-PA reached a good outcome at three months (mRS 0–2) more often than patients who did not receive t-PA (18 (47.4%) vs. two (13.3%), *p* = 0.028). There were no differences in the rate of symptomatic bleeding rate and final infarct volumes.

## 4. Discussion

One of the main findings of our study is that bridging with intravenous t-PA in patients with acute ischemic stroke caused by TIM occlusion treated with EVT is associated with a more favorable three-month outcome. In addition to analyzing our own data, we also performed a systematic review of literature regarding outcomes in studies of patients with acute TIM occlusion receiving EVT (Table 4). 

Among a total of 547 patients reported in four other studies comparable to ours, the median admission NIHSS ranged from 15–18, with a successful recanalization (TICI 2b and 3) rate of 62–79%. The outcome at three months was good (mRS 0–2) in 32-52% of the patients. The findings in our study are comparable regarding the number of patients (*n* = 53), admission NIHSS (median 21), and outcomes. However, there are some differences as well. Our patients were older and we had only two cases (4%) with M2 occlusion, whereas other studies reported a rate of 10–11.1% [10,11]. Acute stenting was performed only in 13% of our patients, which is significantly lower than that reported by the other studies. This is related to the high proportion of patients with cardioembolic stroke (50%) and the fact that we defined TIM as inclusive of intracranial ICA occlusions as well. However, a pooled analysis demonstrated good efficacy of EVT in 122 patients with TIM occlusion, regardless of acute stent placement [12]. Furthermore, emergency stenting of ICA with the use of abciximab may potentially be associated with a higher rate of sICH [23].

Previous published studies have demonstrated a mortality rate of 19–39% [10,11,24], which is comparable to ours (28%). In-hospital mortality occurred at a median of 2.5 days, reflecting the devastating nature of malignant MCA infarction that results in 80% mortality [25]. Indeed, an infarct volume of 220 mL or more is predictive of severe brain edema that peaks from day one [26]. Of note, our patients with a bad outcome had a median FIV of 164 mL (cm^3^).

Successful reperfusion was achieved in 68%, consistent with previous studies [10]. The incidence of sICH was 28%, also comparable with previous studies that detected a prevalence of 4.2–22%. However, not all sICH resulted in in-hospital death, with four surviving patients in our population. 

A study investigating outcomes after thrombolysis alone compared to thrombolysis with add-on EVT during the first 4.5 hours after stroke demonstrated more favorable outcomes with the latter approach in terms of mRS (0–2) at three months (35.0 vs. 18.4%; adjusted OR (aOR) 2.79; 95% CI 1.66–4.67) and mortality rates (17.9 vs. 35.4%; aOR 0.24; 95% CI 0.13–0.42) [16]. Our patients treated with t-PA and EVT had a better chance of favorable outcome, comparable with a previous report [11]. Systemic thrombolysis may on the one hand serve as an adjunct for EVT by altering clot properties and thus aiding mechanical recanalization. On the other hand, t-PA may dissolve harder-to-reach clots and additional microemboli.

Obvious limitations of our study are its retrospective nature, the relatively small number of patients, and the heterogeneity of procedures. However, it reflects practice in a real-world scenario in two tertiary European centers.

## 5. Conclusions

Our findings provide evidence that t-PA is of added value in EVT-treated patients with tandem ICA and MCA occlusion and improves early outcome. Eventually, a randomized-controlled clinical trial which aims to enroll 404 patients will further clarify the role of adjunct t-PA in TIM [33].

## Figures and Tables

**Table 1:** Baseline clinical characteristics of patients with favorable and unfavorable outcomes following EVT for tandem internal carotid and middle cerebral artery occlusion.

Characteristics	All *n* = 53	Favorable Outcome (mRS 0–2) *n* = 20 (37.7%)	Unfavorable Outcome (mRS 3–6) *n* = 33 (62.3%)	*p* Value
Age, median, IQR	75.9 (64.6–82.6)	66.5 (60.5–78.5)	79.0 (72.0–84.0)	0.011
Male gender (%)	28 (52.8)	12 (60.0)	16 (48.5)	0.567
Premorbid mRS 2–4 (%)	5 (9.3)	1 (5.0)	9 (27.3)	0.295
Admission NIHSS score IQR	20 (16–23)	18 (16–22)	22 (16–23)	0.317
Discharge NIHSS score (*n* = 39) IQR	19 (10–18)	9 (4–16)	15 (10–18)	<0.001
TOAST Classification				
Cardioembolism and unknown (%)	38 (71.6)	11 (55.0)	27 (81.8)	0.058
Large artery	9 (17.0)	4 (20)	5 (15.2)	
Other causes (%)	6 (11.3)	5 (25.0)	1 (30.0)	
Risk Factors				
TIA/stroke (%)	11 (20.7)	4 (20.0)	6 (18.2)	1.000
Peripheral arterial occlusive disease (%)	9 (16.9)	2 (10.0)	7 (21.2)	0.456
Atrial fibrillation (%)	21 (39.6)	8 (40.0)	13 (39.4)	1.000
Diabetes (%)	17 (13.2)	3 (15.0)	4 (12.1)	1.000
Arterial hypertension (%)	32 (60.4)	9 (45.0)	23 (69.7)	0.090
Carotid stenosis ≥50% (%)	12 (22.6)	5 (25.0)	7 (21.1)	0.748
Glucose (*n* = 51), mg/dL	129 (115–146)	122 (106–136)	130 (117–161)	0.050
Cholesterol (*n* = 45), mg/dL	158 (132–184)	175 (147–208)	143 (123–171)	0.026
LDL (*n* = 43), mg/dL	93 (72–116)	108 (92–131)	82 (65–102)	0.011
Erythrocyte count (*n* = 47)	4.5 (4.1–4.8)	4.6 (4.4–4.8)	4.2 (3.9–4.6)	0.017
Thrombocyte count (*n* = 47)	229 (188–299)	264 (198–341)	214 (170–260)	0.061
Antithrombotic Treatment Before Stroke				
Aspirin (%)	16 (30.2)	6 (30.0)	9 (27.3)	1.000
Anticoagulant (%)	9 (16.9)	4 (20.0)	5 (15.1)	0.715

Abbreviations: EVT—endovascular treatment; NIHSS—National Institutes of Health Stroke scale; mRS—modified Rankin scale; TOAST—Trial of Org 10172 in Acute Stroke Treatment. Values in parentheses refers to interquartile range (IQR).

**Table 2:** Radiological characteristics of 53 patients with favorable and unfavorable outcomes following EVT for tandem internal carotid and middle cerebral artery occlusion.

Radiological characteristics	Unadjusted Data (*n*, Mean ± SD, or Median, Interquartile Range)			
	All, *n* = 53	Favorable Outcome (mRS 0–2) *n* = 20	Unfavorable Outcome (mRS 3–6) *n* = 33	*p* Value
Vessel Characteristics				
MCA M1 occlusion	51 (96.2)	18 (90.0)	33 (100.0)	0.138
MCA M2 occlusion	2 (3.8)	2 (10.0)	0 (0.0)	
ASPECTS	8 (7–9)	9 (8–9)	8 (7–9)	0.248
Leptomeningeal Collateralization				
Absent or less on the affected side	39 (82.9)	14 (77.8)	25 (86.2)	0.407
Equal to unaffected side	8 (17.0)	4 (22.2)	4 (13.8)	
Treatment				
t–PA use (%)	37 (69.8)	18 (90.9)	19 (57.6)	0.015
Time to first imaging (min)	85 (64–108)	84 (58–103)	85 (64–113)	0.380
Time to needle (min, *n* = 36)	110 (90–130)	104 (85–130)	120 (94–130)	0.419
Time to vessel (min)	181 (160–227)	186 (163–235)	175 (142–226)	0.388
Time to recanalization (min)	276 (207–323)	278 (213–309)	273 (206–337)	0.962
EVT intervention time	76 (42–116)	74 (40–89)	76 (42–124)	0.344
Acute ICA stenting (%)	7 (13.2)	4 (20.0)	3 (9.1)	0.400
TICI Outcome				
0–2a (%)	17 (32.1)	2 (10.0)	15 (45.4)	0.014
2b–3 (%)	36 (67.9)	18 (90.0)	18 (54.5)	
		Symptomatic hemorrhage (%)		
	14 (28.0)	4 (21.0)	10 (32.3)	0.288
		Final infarct volume in cm^3^ (*n* = 50)		
	58.1 (17.9–202.9)	24.4 (3.9–41.7)	163.9 (52.3–315.5)	<0.001

Abbrevations: ASPECTS—Alberta stroke programme early CT score; t-PA—tissue-Plasminogen activator; ICA—internal carotid artery.

**Table 3:** Predictors of a good outcome after 3 months in 50 patients treated with mechanical thrombectomy due to internal carotid and middle cerebral artery occlusion.

Predictors of outcome	Odds Ratio	95% CI	*p*
Age per point increase	0.889	0.805–0.981	0.020
NIHSS per point increase	1.121	0.950–1.324	0.175
Successful recanalization TICI 2b and 3	4.876	0.424–55.988	0.203
Final infarct volume above 25th percentile	0.021	0.001–0.332	0.005
t–PA administered	12.045	1.004–144.392	0.049

**Table 4:** Review of previous studies on endovascular treatment of tandem occlusion of internal carotid/middle cerebral artery.

Studies	*n*	Age, Years, Range	NIHSS at Admission	IV t–PA (%)	Carotid Stent	Successful Recanalization	ICH	Mortality	Good Functional Outcome at 3 Months
Current Study	53	75.9 (64.6–82.6)	18 (16–22)	37 (69.8)	7 (13.2)	36 (67.9)	14 (28.0)	15 (28.3)	20 (37.8)
Sallustio et al. [10]	72	65.6 ± 12.8	19 ± 2.9	39 (54.1)	35 (48.6)	46 (64.0)	9 (12.5)	23 (31.9)	23 (31.9)
Heck et al. [24]	23	70 (45–86)	17 (9–25)	12 (52.0)	23 (100.0)	17 (74.0)	5 (22.0)	9 (39.0)	12 (52.0)
Malik et al. [27]	77	63.4 ± 10.9	14.8 ± 5.4	-	77 (100.0)	58 (75.3)	8 (10.4)	19 (24.7)	32 (41.6)
Behme et al. [11]	170	64 (25–88)	15 (12–19)	122 (72.0)	180 (100.0)	130 (77.0)	15 (9.0)	32 (19)	61 (36.0)
Cohen et al. [28]	24	66 (51–77)	20.4 (14–28)	10 (41.6)	24 (100.0)	19 (79.0)	6 (25.0)	2 (8)	13 (76)
Lockau et al. [29]	37	63 (36–89)	17 (3–30)	20 (54.1)	37 (100.0)	27 (73.0)	4 (10.8)	7 (18.9)	17 (45.9)
Puri et al. [30]	28	58.7 (30–83)	18 (15–22)	8 (27.3)	28 (100.0)	20 (71.4)	2 (7.1)	4 (14.3)	11 (39.3)
Lescher et al. [31]	39	68 (38–92)	14 (6–20)	20 (74.0)	39 (100.0)	25 (64.0)	4 (10.0)	4 (10.0)	14 (36)
Stampfl et al. [32]	24	67 (49–83)	18 (15–22)	NA	24 (100.0)	15 (62.5)	1 (4.2)	4 (16.6)	7 (29.2)

Numbers in parentheses are percentages, except age in years, which is given as median with interquartile range or mean with standard deviation. NIHSS—National Institutes of Health Stroke Scale; t-PA—intravenous thrombolysis with recombinant tissue plasminogen activator; ICH—intracerebral hemorrhage; NA—not available. Successful recanalization defined as thrombolysis in cerebral infarction (TICI) grade 2b or 3.
